# Efficacy of treatment with tumor-infiltrating lymphocytes as adoptive cell therapy: an integrative review

**DOI:** 10.31744/einstein_journal/2024RW0935

**Published:** 2024-11-22

**Authors:** Lucas Marques Soares da Silva, Eleni Solange de Brito Gomes, Julia Hailer Vieira, Murilo Porfírio de Aguiar, Saulo Fernando Moreira da Silva, Marcia Antoniazi Michelin

**Affiliations:** 1 Universidade Federal do Triângulo Mineiro Instituto de Pesquisa em Oncologia Uberaba MG Brazil Instituto de Pesquisa em Oncologia, Universidade Federal do Triângulo Mineiro, Uberaba, MG, Brazil.; 2 Universidade Federal do Triângulo Mineiro Uberaba MG Brazil Universidade Federal do Triângulo Mineiro, Uberaba, MG, Brazil.

**Keywords:** Lymphocytes, tumor-infiltrating, Neoplasms, Immunotherapy, Immunotherapy, adoptive

## Abstract

**Objective:**

This integrative review article examines the efficacy of adoptive cell therapy using tumor-infiltrating lymphocytes, with a particular focus on the treatment of melanomas and other solid tumors.

**Methods:**

The methodology encompasses theme definition, comprehensive database searches, and a critical review of pertinent literature. Of the 1,947 articles initially identified, 15 were meticulously selected based on stringent inclusion and exclusion criteria.

**Results:**

The findings suggest that tumor-infiltrating lymphocytes-based therapy is particularly effective in treating metastatic melanomas, as noted by its tailored approach and substantial potential. However, the applicability of these findings to other solid tumor types remains limited.

**Conclusion:**

This review indicates that adoptive cell therapy using tumor-infiltrating lymphocytes demonstrates efficacy, especially in the treatment of metastatic melanoma, and shows considerable promise for treating solid tumors.

## INTRODUCTION

Cancer is a mounting global public health concern with notable incidence and mortality rates. In Brazil, an estimated 704,000 new cancer cases are expected between 2023 and 2025.^(1)^ This situation underscores the necessity for efficacious treatments that go beyond traditional modalities such as radiotherapy, chemotherapy, and surgery, with the aim of improving patient quality of life and extending survival, potentially culminating in complete tumor remission. In this context, immunotherapy has emerged as a promising avenue for integrating clinical strategies into customized cancer treatments. Presently, an array of immunotherapeutic options are available, including checkpoint inhibitors,^(2)^ cytokines such as interferon-alpha,^(3)^ dendritic cell vaccines,^(4)^ CAR-T cell therapy,^(5)^ and adoptive cell therapy with tumor-infiltrating lymphocytes (TILs).^(6)^ Adoptive cell therapy involves the cultivation of TILs, extracted from tumor tissues, and reintroduction into patients to establish personalized and autologous therapy. Reinfusion is often conducted in conjunction with Interleukin-2 (IL-2) to facilitate the expansion of these cells and enhance antitumor response.^(7,8)^

Therefore, this study focused on the therapeutic potential of TILs in clinical scenarios via an integrative review of the literature to scrutinize the available evidence on their effectiveness in cancer immunotherapy.

## METHODS

This study was an integrative review executed through distinct phases:

Step 1 (Problem formulation): identification of the topic and formulation of the research question relevant to the health field; Step 2 (Identification): development of the search strategy and definition of the inclusion and exclusion criteria (search strategy); Step 3 (Screening): selection and categorization of the relevant studies; critical appraisal of the selected articles (critical appraisal of the search results); Step 4, 5 and 6 (Included): synthesis and interpretation of the results (summary of search results, data extraction and analysis); and Step 7: presentation of the results (conclusion and implications).

**Figure 1:**
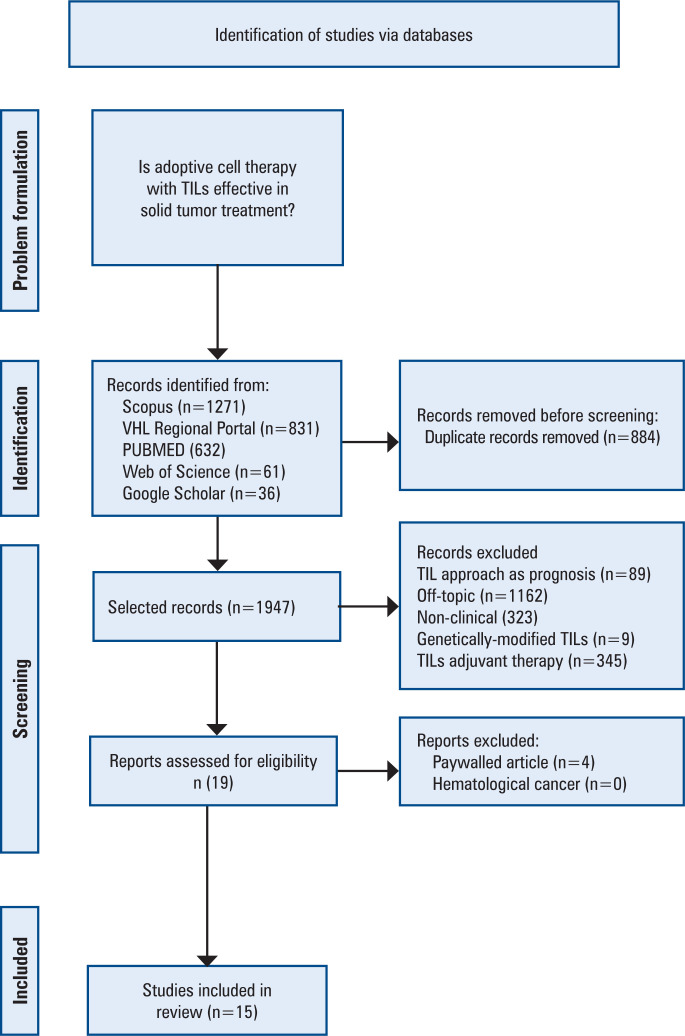
Study selection process Information was synthesized by extracting relevant results regarding the treatment of solid tumors with TILs and conclusions from each article. The data were standardized to compile a coherent and uniform table of results TILs: tumorinfiltrating lymphocytes.

Source: Prepared by the authors, based on the PRISMA Protocol of 2020.

Briefly, the research, conducted between May and December 2023, centered around the primary question (Step 1): "Is adoptive cell therapy with TILs effective in solid tumor treatment?" The Scopus, VHL Regional Portal (https://search.bvsalud.org/portal/advanced/lang=en), PUBMED, Web of Science, and Google Scholar databases were searched. Health Sciences Descriptors (DeCS) and Medical Subject Headings (MeSH) were employed to tailor the Boolean operator sequence and application to each database specification (Step 2). The details of this strategy are provided in the Supplementary Material. In total, 2,831 articles published between 2018 and 2023 in English and Portuguese were included (Step 2). Of these, 884 were excluded because of duplication, leaving 1,947 articles for the initial screening. During this phase, each abstract underwent an independent, blinded evaluation by all authors on the Rayyan platform to mitigate cross-influence. The exclusion criteria in the screening phase (Step 3) included articles not addressing TILs, studies on TILs as a prognostic factor, non-clinical research, use of genetically modified TILs, and combined TIL therapies. After exclusion, 19 articles were identified for in-depth analysis. Ultimately, 15 of the 19 articles were selected for this study (Steps 4, 5, and 6). The exclusion criteria in this final phase were the unavailability of complete text from the university and articles not involving patients with solid tumors. The methodology is illustrated in detail in figure 1.

## RESULTS

Among the 15 articles on the efficacy of TILs in adoptive cell therapy selected in this study, 13 (86.67%) focused on various types of melanoma, while two articles (13.33%) addressed other types of cancer, with one dedicated to ovarian cancer and the other encompassing multiple types including colorectal, pancreatic, breast, ovarian, and cervical cancers. All articles indicated the effectiveness of TILs in cancer treatment. Table 1 presents the detailed findings from the included studies related to the use of TILs.

**Table 1:** Studies related to the use of tumor-infiltrating lymphocytes that are eligible for the review

Authors/ Citation/ Year	Type of tumor	Study design	Results	Conclusion
Rohaan et al.^(8)^ 2022	Advanced melanoma	Multicenter, open-label, phase 3 trial	1) 164 patients were randomized, with 84 receiving TIL infusion treatment and 84 treated with Ipilimumab 2) The TIL infusion group had a median progression-free survival of 7.2 months, compared to 3.1 months in the Ipilimumab group 3) Median overall survival was 25.8 months in the TIL group versus 18.9 months in the Ipilimumab group	The progression-free survival in patients with advanced melanoma was significantly longer among those who received TIL therapy compared to those who received Ipilimumab
Hall et al.^(9)^ 2023	Metastatic melanoma	Retrospective study	1) Infused TIL failed to produce IFNg in vitro in response to autologous tumor 2) Demonstrated a multifaceted effector response from the TILs, with the release of the cytolytic enzyme granzyme B and critical anti-tumor cytokines, IFNg, and TNFα	1) The study supports the importance of prospectively identifying neoantigen-specific CD4+ T cells in TIL products as potent tumor effectors
Chesney et al.^(10)^ 2022	Advanced melanoma	Phase 2 clinical trial	1) Results showed an objective response rate (ORR) of 31.4%, while the median duration of response was not reached at a median study follow-up of 27.6 months, with 41.7% of the responses maintained for ≥ 18 months. 2) The median overall survival and progression-free survival were 13.9 and 4.1 months, respectively 3) The most common grade 3/4 treatment-emergent adverse events (≥ 30%) were thrombocytopenia (76.9%), anemia (50.0%), and febrile neutropenia (41.7%)	1) The treatment demonstrated clinically significant activity in heavily pre-treated patients with advanced melanoma and high tumor burden 2) Single lifileucel TIL cell therapy was beneficial in patients with treatment limitations in ICI refractory disease, with durable responses and a favorable safety profile
Saberian et al. ^(11)^ 2021	Metastatic melanoma	Randomized phase II trial	1) A patient who exhibited a very long progression-free survival of over 90 months had the majority (> 50%) of CD8+ TILs in their infusion product. Additionally, patients who received an infusion of a TIL product containing a high proportion of CD8+ TILs lived longer (OS above 5 years). In general, patients with > 50% CD8+TILs in their infusion product tended to have a longer overall survival	1) TIL therapy alone shows greater persistence of MART-1 TILs than combined TIL+DC therapy 2) The lack of benefit from DC co-vaccination may be attributed to the chosen antigen, as MART-1 reactive TILs naturally occur at high frequency in patients with melanoma and have been shown to persist post-infusion without DC vaccine aid 3) Study limitation is the focus on a single tumor antigen and DC co-vaccination approaches
Saint-Jean et al.^(12)^ 2018	Advanced melanoma	Monocenter retrospective study	1) ACT yielded clinical benefit in 4 patients (including 2 with objective responses) undergoing third-line therapy or beyond, with a complete response in patient 4, partial response in patient 2, and stable disease in patients 8 and 10; out of 10 patients 2) Regarding ACT toxicity, no grade 4 side effects were reported; only grade 3 nausea and vomiting were observed. All other adverse events were grade 1 or 2, associated with IL-2 injections, not TILs	1) In summary, the results showed that ACT without a lymphodepleting regimen and with low doses of subcutaneous IL-2 was safe in heavily pre-treated patients with advanced melanoma 2) A higher percentage of CD4+ CD25+ CD127lowFoxp3+ T cells in the infused TIL population was associated with significantly shorter OS 3) Combining ACT with checkpoint inhibitors could potentiate the TIL effect, countering local immunodeficiency
Mehta et al.^(13)^ 2018	Metastatic melanoma with and without brain metastases	Retrospective study	1) Durability of Responses: the durability of objective responses was shorter in patients with brain metastases, compared to those without a history of brain disease 2) Disease Progression: Patients with untreated brain metastases were more likely to progress first at the untreated disease site (56%) 3) Disease-Free Progression: Patients without brain metastases showed longer PFS compared to those with untreated brain metastases. This reflects the tendency of progression for untreated brain metastasis and the need for additional treatment post-TIL therapy	TILs can migrate to the brain and safely mediate complete, durable regression of brain disease in some patients with brain metastasis. While the treatment showed potential for regressing brain disease in some cases, most patients with untreated brain disease required additional therapy post-TIL. These findings underscore the importance of considering brain disease presence when interpreting and planning clinical trials for patients with advanced melanoma
Kristensen et al.^(14)^ 2022	Melanoma	Prospective observational study	1) A higher presence of CD8+ T cells targeting novel markers in TIL was linked to increased survival. Furthermore, CD8+ T cells recognizing these novel markers in the infusion product were only found in patients who responded to TIL treatment	This study highlights the critical importance of neoantigens recognized by specific T cells (NARTs) in the clinical outcomes of TIL-ACT therapy. Moreover, it provides a comprehensive analysis of novel markers recognized by T cells in this therapeutic setting
Khammar et al.^(15)^ 2022	Advanced melanoma	Multicenter, randomized clinical phase III trial	1) Overall survival showed no significant difference, however, the TIL-treated group tended to have a higher survival rate 2) The TIL-treated group showed a longer disease-free survival duration compared to the Control Group	The study demonstrated that adjuvant treatment with TIL + IL2 in patients with ulcerated lesions resulted in longer, though not significantly different, disease-free survival than in those treated with IL2 alone
Hirai et al.^(16)^ 2021	Metastatic melanoma	Open-label, single-arm, pilot study	1) Expanded TILs were predominantly CD8+ T cells in 2 patients, and CD4+ T cells in 1 patient 2) Patient 1: lesions remained stable for 9.3 months after TIL infusion. Patient 2: discontinued treatment due to pain. Patient 3: experienced short-term tumor regression	The TIL-ACT protocol demonstrated clinical benefit in two patients, a short PR in patient 3, and a long SD in patient 1, both of whom had previously failed third-line or beyond treatment with anti-PD-1 or CTLA-4 Ab
Pedersen et al.^(17)^ 2018	Ovarian cancer	Pilot study	1) Six of the eleven patients enrolled underwent treatment, demonstrating successful TIL expansion. TIL therapy resulted in stable disease for most of the patients, with target lesion reduction and fluctuations in CA-125 levels Immunological analysis revealed specific anti-tumor reactivity and varied T-cell differentiation stages. Despite some adverse effects, the treatment was generally well-tolerated, indicating the therapeutic potential of TILs in selected cancer cases	The study showed that TIL therapy in patients with metastatic ovarian cancer is a feasible approach with manageable toxicities. However, overall efficacy was limited despite some clinical activity, like temporary disease stabilization, suggesting the need to combine this therapy with immune checkpoint inhibitors to enhance therapeutic outcomes
Forget et al.^(18)^ 2018	Metastatic melanoma	Clinical trial	1) The study found that patients with metastatic patients treated with TIL ACT therapy post anti-CTLA4 exposure had a lower overall response rate and median overall survival. TIL persistence, tumor mutational burden, and recognition of autologous tumors did not significantly impact outcomes. However, reduced infused TIL quantity and shorter treatment response duration were noted in patients previously exposed to anti-CTLA4. The study also identified IL-9 as a predictive factor for a favorable response to TIL ACT therapy	TIL therapy in patients with metastatic melanoma exhibits clinical efficacy but is less effective in patients previously treated with anti-CTLA4, showing reduced overall responses and overall survival
Creasy et al.^(19)^ 2022	Metastatic melanoma	Observational study	1) A positive correlation was observed between higher overall survival and a higher burden of non-silent mutations; however, no correlation was found with progression-free survival or response 2) Elevated PDE1C, RTKN2, and NGFR were detected in patient samples and were associated with therapeutic response, improved progression-free survival, and enhanced overall survival. 3) ELFN1 was consistently elevated in patients who did not respond to therapy and had low overall and progression-free survival	Only ELFN1 was associated with poor progression-free and overall survival, and a lack of clinical response
van den Berg et al.^(20)^ 2020	Metastatic melanoma	Phase I/II feasibility study	1) Of 10 treated patients, two achieved a complete response, three had a partial response, one exhibited stable disease for 2 months, and four experienced progressive disease 2) Patient 3 remains disease-free for over 9 years post-TIL therapy 3) Patient 4 exhibited partial remission one month post-TIL infusion 4) Patient 8 showed mesenteric lymph node metastases and PR one month post-TIL infusion. Ten months post-TIL infusion, the patient achieved CR and currently maintains a continuous complete response for 7 years post-therapy	The study demonstrated a 50% overall response rate, including a durable 20% complete response rate in this small clinical trial, with specific T-cell reactivity to neoantigens observed in all analyzed infusion products
Levi et al.^(21)^ 2022	Metastatic melanoma	Observational study	1) Patients responding to autologous TIL treatment had more identified neoantigens than non-responders in both anti-PD-1 naïve and experienced cohorts 2) A slightly higher median CD8 TIL density was observed in patients with anti-PD-1 experience compared to those without prior anti-PD-1 treatment	These results indicate that patients with progressive disease post-anti-PD-1 blockade treatment had a lower frequency of neoantigen recognition in their cultured TIL samples, persisting even when accounting for differences in tumor mutational burden, and lower response rates to subsequent adoptive cell therapy
Kim et al.^(22)^ 2022	Ovarian, colorectal, pancreatic, breast, and cervical cancer	Clinical trial	1) Ovarian Cancer: Partial response observed in one patient, indicating limited efficacy in this subset of patients 2) Colorectal Cancer: Absence of significant responses, suggesting limited efficacy of TIL therapy 3) Pancreatic Cancer: Lack of specific details precludes conclusive efficacy assessment 4) Breast Cancer: Partial response in one patient, indicating potential efficacy in this restricted patient group 5) Cervical Cancer: Unspecified results, hindering the assessment of TIL efficacy	The study concludes that TIL therapy, specifically targeting p53 mutations, could be a promising strategy in advanced cancer treatment. However, challenges such as low frequency and limited persistence of tumor-specific neoantigen-reactive TILs, like p53 mutations, need to be addressed to enhance therapy efficacy

## DISCUSSION

Evaluation of immunotherapy efficacy involves the assessment of tumor progression and immunological markers. Kim et al. showed that TIL immunotherapy effectively combats various solid tumors.^(22)^ Levi et al. demonstrated a significant tumor reduction in patients with metastatic melanoma.^(21)^ Hall et al. highlighted that TIL infusion produced high levels of IFN-γ, suggesting Th1 activation.^(9)^ T helper cells (CD4+), specifically, Th1 cells with signature cytokines like IFN-γ, are pivotal for effective anti-tumor responses, as they activate transcription factors increasing IL-12 secretion and CD8+ T cell cytotoxicity.^(21)^ However, van Den Berg et al. found a reduced efficacy of TIL treatment in patients previously treated with checkpoint immunotherapy.^(20)^ Additionally, Forget et al. noted lower survival in patients who received checkpoint inhibitors before TIL therapy than in those who received TIL-only treatments.^(18)^ In contrast, Rohaan et al. compared TIL immunotherapy and monoclonal antibody therapy and showed longer disease-free survival and higher survival rates with TILs,^(8)^ supporting Khammari et al.^(15)^ Chesney et al. also reported significant overall survival with TIL therapy, albeit with notable adverse effects.^(10)^

IL-2 in TIL therapy can induce toxicity, as observed by Pedersen et al., who found that significant clinical responses were accompanied by adverse effects.^(17)^ Pedersen et al. also noted that patients with a Treg TIL phenotype had lower survival rates. Creasy et al. found reduced efficacy of TIL therapy in patients with ELFN1 gene mutations, although methylation of these mutated genes improved responses.^(19)^ Mehta et al. demonstrated objective responses in metastatic patients treated with TIL therapy, with higher efficacy in non-metastatic patients.^(13)^ Saberian et al. and Hirai et al. highlighted the improved clinical responses in patients receiving phenotyped CD8+ cytotoxic TILs.^(11,16)^ Kristensen et al. emphasized the benefits of infusing CD8+ T-cells with a broader range of targetable tumor antigens.^(14)^

## CONCLUSION

This review demonstrates that adoptive cell therapy with tumor-infiltrating lymphocytes is particularly effective in treating metastatic melanoma and shows considerable promise for treating solid tumors. However, it is crucial to note that the scope of our analyzed data was limited, as it did not encompass all solid cancer types. This limitation restricts our ability to extend the efficacy of therapy to a wider array of solid tumors.
